# Rac1 Constrains Memory Consolidation

**DOI:** 10.1523/ENEURO.0448-25.2026

**Published:** 2026-04-03

**Authors:** Gabriel Fernandes Borges, Beatriz do Nascimento Pinheiro Moura, Thays Alves Monteiro, Andressa Radiske, Martín Cammarota, Maria Carolina Gonzalez

**Affiliations:** ^1^Edmond and Lily Safra International Institute of Neuroscience, Santos Dumont Institute, Macaiba, RN 59280-000, Brazil; ^2^Memory Research Laboratory, Brain Institute, UFRN, Natal, RN 59078-970, Brazil

**Keywords:** 1A-116, hippocampus, memory persistence, object recognition memory, short-term memory

## Abstract

Ras-related C3 botulinum toxin substrate 1 (Rac1) is a small GTPase that regulates actin cytoskeleton dynamics and synaptic plasticity. Rac1 has been implicated in active forgetting, but whether it also constrains the consolidation of new memories remains unclear. Here we show that systemic administration of the Rac1 inhibitor 1A-116 after training in the novel object recognition task markedly extends memory persistence in rats. A single post-training injection of 1A-116 enhanced recognition memory for at least 28 d without altering locomotor- or anxiety-related behaviors. When given after a brief, subthreshold training session that normally supports only short-term memory, 1A-116 enabled long-term retention that required hippocampal protein synthesis. This promnesic effect was time-dependent, independent of sex, and consistent with Rac1 acting as a negative regulator of memory consolidation rather than merely promoting forgetting. These findings indicate that Rac1 activity after learning limits the consolidation process itself, functioning as a molecular brake on recognition memory stabilization, and suggest that its inhibition may represent a therapeutic avenue to enhance cognitive durability in both healthy and pathological conditions.

## Significance Statement

Memory persistence is shaped by both consolidation and active forgetting, yet the molecular constraints that determine how long a memory lasts remain partially understood. We demonstrate that Rac1, a small GTPase involved in actin remodeling, serves as a negative regulator of hippocampal-dependent recognition memory consolidation. Pharmacological inhibition of Rac1 after learning not only enhances retention but also enables long-term memory formation from subthreshold training through a hippocampal protein synthesis-dependent mechanism. These findings identify Rac1 activity as a molecular brake on memory stabilization and suggest that its inhibition may enhance cognitive persistence and resilience against age- or disease-related decline.

## Introduction

The persistence of memory depends on a delicate balance between consolidation and forgetting (Dalto et al., 2025). The molecular mechanisms that support consolidation, including protein synthesis, gene transcription, and synaptic remodeling, have been extensively characterized, but the factors that actively limit consolidation remain less understood.

Ras-related C3 botulinum toxin substrate 1 (Rac1), a member of the Rho family of small GTPases, is a central modulator of structural plasticity. Through downstream effectors such as PAK, LIMK1, and cofilin, Rac1 orchestrates actin polymerization and dendritic spine morphology (Wiens et al., 2005; Haditsch et al., 2009). Rac1 activation is tightly coupled to NMDA receptor signaling and calcium influx (Tolias et al., 2005), positioning it as a pivotal switch in activity-dependent synaptic remodeling. However, excessive or sustained Rac1 activation can destabilize dendritic spines, impair long-term potentiation (LTP), and promote memory decay (Liu et al., 2016; Cui et al., 2021).

Initial studies in *Drosophila* demonstrated that Rac1 contributes both to passive memory decay and to interference-induced retrieval impairment (Shuai et al., 2010; Davis and Zhong, 2017). Subsequent work in mammals partially extended these findings, showing that hippocampal Rac1 hyperactivation shortens memory retention (Wu et al., 2019), whereas pharmacological or genetic inhibition of Rac1 prolongs recognition memory persistence (Liu et al., 2016; O’Leary et al., 2024). Taken together, these observations led some to suggest that Rac1 promotes a physiological process of active forgetting involving engram degradation and memory erasure (Davis and Zhong, 2017). This view relies largely on evidence from *Drosophila* showing that the promnesic effects of Rac1 inhibition occur independently of memory formation mechanisms (Shuai et al., 2010), thereby excluding the alternative explanation that Rac1 inhibition enhances memory by strengthening consolidation (Medina, 2018). This distinction is critical. Although some neural mechanisms implicated in forgetting are engaged at the time of initial learning and unfold on a time course similar to that of consolidation, active forgetting is defined as a process that regulates memory decay and suppresses previously consolidated, yet behaviorally irrelevant, long-term memories (Davis and Zhong, 2017). In contrast, restricted consolidation refers to gating mechanisms that limit the stabilization of newly encoded information blocking the conversion of short-term memories into long-term memories and/or increasing long-term memory persistence (Abel and Kandel, 1998).

Object recognition memory (ORM) enables animals to distinguish between familiar and novel objects, supporting the encoding and retrieval of episodic-like memory (Rossato et al., 2026). The present study was designed to determine whether Rac1 activity constrains ORM consolidation or mediates its active forgetting. To do that, we employed the novel object recognition (NOR) task, a robust single-trial incidental learning paradigm that captures key aspects of episodic memory (Ennaceur and Delacour, 1988; Aggleton and Nelson, 2020) and depends on hippocampal plasticity (Gonzalez et al., 2019, 2021). We administered the Rac1 inhibitor 1A-116 systemically after training and evaluated ORM persistence across varying training strengths and intervals, in both sexes. In addition, we examined whether the resulting long-term ORM required hippocampal protein synthesis, as expected for canonical consolidation (Rossato et al., 2007). Our results reveal that Rac1 activity immediately after learning acts as a molecular constraint on memory consolidation and that its inhibition releases this brake, enabling protein synthesis-dependent stabilization of ORM lasting several weeks.

## Materials and Methods

### Animals

We used young adult (4–5 months old) or middle-aged (14–16 months old) Wistar rats of both sexes (*Rattus norvegicus*; RRID: RGD_13508588). Males and females were housed separately in groups of 3–4 per cage in the institutional animal facility. Animals were kept in ventilated polypropylene cages under controlled temperature (22–24°C) with *ad libitum* access to food (standard rat chow) and water. The light/dark cycle was maintained at 12 h (lights on at 7 A.M.), and all experiments were conducted during the light phase. Before the experiments, animals were transported to the experimental room, tail-marked for identification, and handled during 2 min for 2–3 consecutive days. Body weight was recorded 1 d before drug administration. All procedures followed the US National Institutes of Health Guidelines for Animal Care and were approved by the Animal Research Ethics Committee of the Santos Dumont Institute [Comissão de Ética no Uso de Animais do Instituto Santos Dumont (CEUA/ISD)].

### Stereotaxic surgery

Surgeries were conducted under aseptic conditions. Anesthesia was induced with ketamine (10 mg/kg, i.m.) and maintained with isoflurane (1.5–3.5%) in oxygen (1–1.5 L/min). Young adult male rats were bilaterally implanted with 22-gauge stainless steel guide cannulas targeting the CA1 region of the dorsal hippocampus (AP −4.2 mm; ML, ±3.0 mm; DV, −3.0 mm from bregma). Stereotaxic coordinates were based on previous studies and a rat brain atlas (Paxinos and Watson, 2007; Gonzalez et al., 2021). After surgery, animals received meloxicam (0.2 mg/kg, s.c.) for analgesia and were allowed to recover for 7 d before behavioral testing.

### Drugs and infusion procedures

Drug doses were selected based on previous studies and pilot experiments. Anisomycin (100 µg/µl; Sigma-Aldrich/Merck; catalog #A9789; Radiske et al., 2017) was dissolved in acidic saline (pH ∼3), and the pH was adjusted to ∼7.0 with NaOH. The solution was aliquoted and stored at −20°C as a concentrated stock. On the day of the experiment, aliquots were diluted to working concentration in sterile 0.9% saline. 1A-116 (20 mg/kg; kindly provided by Dr. Georgina Cardama, National University of Quilmes) was first dissolved in acidic water (pH 2, adjusted with 100 mM HCl), then neutralized to pH 6 with 100 mM NaOH, and diluted in sterile saline to working concentration. Intra-CA1 injections (1 µl per side) were administered using a Hamilton syringe with infusers fitted into the guide cannulas. Infusers were left in place for one additional minute to minimize backflow.

### Novel object recognition task

Animals were acclimated to the experimental room for 40–60 min under low light (∼15 lux) before testing. Habituation consisted of 20 min daily sessions in the empty arena (gray plywood, 60 cm × 60 cm × 60 cm) for 3 consecutive days. One day after habituation, rats were placed in the training arena for 5 min or 90 s (weak training) in the presence of two identical novel objects (A and A’; training session). Object recognition memory retention was assessed at specific intervals by re-exposing the animals to the arena containing one familiar object A and one novel object B for 5 min (test session; Gonzalez et al., 2022; Rossato et al., 2022). The animals were tested once. Objects were made of metal, glass, or glazed ceramic, and measured approximately 10–17 cm in height and 5–7 cm in width. The objects were placed along the arena's centerline, equidistant from the walls. Between trials, the arena and objects were cleaned (70% ethanol) to eliminate olfactory cues. Pretests confirmed no innate object preference. Behavior was recorded using overhead digital cameras. Object exploration was defined as sniffing or touching the objects with the muzzle or forepaws; sitting, climbing, or rearing on the objects was not considered as exploratory behavior. Subjects were randomly assigned to the experimental groups. All behavioral scoring was performed by experimenters blind to the animals' treatment.

### Plus maze task

The maze consisted of a black MDF cross-shaped apparatus elevated 50 cm above the floor, with two open and two closed arms (45 cm × 10 cm). Closed arms had 30-cm-high walls. Animals were placed in the center of the maze and allowed to explore freely for 10 min. Behavior was recorded by an overhead camera, and the number of entries, time spent, and distance traveled in open arms were quantified.

### Immunofluorescence

Rats received an intraperitoneal injection of vehicle or 1A-116. Ninety minutes later, animals were deeply anesthetized with isoflurane (5% in oxygen, 1.5 L/min) and transcardially perfused with 4% paraformaldehyde (PFA; pH 7.2). Brains were postfixed overnight in 4% PFA, cryoprotected in 30% sucrose for 2–3 d, and coronally sectioned (50 µm) on a cryostat. Sections containing the dorsal hippocampus were washed in PBS, permeabilized with 1% Triton X-100 for 1 h, and blocked in 10% normal goat serum (NGS) in 0.3% PBST for 1 h at room temperature. Sections were incubated overnight at 4°C with primary antibodies against c-Fos (1:1,000; Santa Cruz Biotechnology; RRID: AB_2106783) and NeuN (1:1,000; EMD Millipore; RRID: AB_2298772) in NGS/PBST, followed by 2 h incubation at room temperature with Alexa Fluor 488-conjugated goat anti-rabbit (1:1,000; Life Technologies/Thermo Fisher Scientific; RRID: AB_143165) and Alexa Fluor 594-conjugated goat anti-mouse (1:1,000; Life Technologies/Thermo Fisher Scientific; RRID: AB_2534073). After washing, sections were counterstained with DAPI (1:1,000, Thermo Fisher Scientific, RRID: AB_2629482) and mounted using Fluoromount-G (Thermo Fisher Scientific; RRID: SCR_015961). Images were acquired with a Zeiss Axio Imager Z2 microscope (20× objective magnification, RRID: SCR_018856). Regions of interest were selected using ImageJ, and c-Fos-positive cells were quantified with Ilastik 1.4.0 (Berg et al., 2019).

### Data analysis

Exploration times during the test session were used to calculate a discrimination index, defined as follows: (time exploring the novel object − time exploring the familiar object) / (time exploring the novel object + time exploring the familiar object). The discrimination index was used as a measure of discrimination between familiar and novel objects, where values near zero indicate no preference, whereas positive values denote a preference for the novel object. Animals that exhibited total exploration time <10 s in either the TR or TEST sessions (∼4%) were excluded from further analyses to avoid possible confounding effects resulting from insufficient exploration. Statistical analyses were performed using GraphPad Prism 8 software (RRID:SCR_002798). Significance was set at *p* < 0.05. The Shapiro–Wilk test was used to assess the normality of data distribution. Discrimination index data were analyzed using a one-sample *t* test with a theoretical mean of 0. Group comparisons were conducted using unpaired *t* tests (with Welch's correction when homogeneity of variance was violated), Mann–Whitney test, or one-way or two-way ANOVA, as appropriate. Effect sizes and their associated uncertainty were calculated using DABEST (Ho et al., 2019), available at https://www.estimationstats.com. Total exploration times at TEST were compared using unpaired *t* tests, unpaired *t* tests with Welch's correction, or two-way ANOVA, as appropriate (Table 1).

**Table 1.** 1A-116 does not affect total exploration time

	Experimental groups		*n*	Total exploration time (s)		Statistical analysis	
				Mean	SEM		
Figure 1*C*	1 d	VEH	7	51.88	06.71	ns	*F*_(1,26)_ = 0.69, *p* = 0.41 for interaction *F*_(1,26)_ = 0.06, *p* = 0.79 for treatment *F*_(1,26)_ = 2.22, *p* = 0.14 for test session
		1A-116	7	57.43	05.31		
	28 d	VEH	8	48.57	03.06	ns	
		1A-116	8	45.66	04.91		
Figure 2*B*		VEH	8	81.63	11.15		*t*_(14)_ = 1.84, *p* = 0.08
		ANI	8	55.47	08.73		
Figure 2*C*	3 h	VEH	8	64.95	09.20	ns	*F*_(3,54)_ = 0.04, *p* = 0.98 for interaction *F*_(1,54)_ = 1.53, *p* = 0.22 for treatment *F*_(3,54)_ = 9.88, *p* < 0.0001 for test session
		1A-116	8	55.38	06.15		
	6 h	VEH	8	91.34	08.86	ns	
		1A-116	8	82.55	07.39		
	9 h	VEH	8	97.41	09.71	ns	
		1A-116	8	89.63	11.84		
	1 d	VEH	7	57.60	04.82	ns	
		1A-116	7	54.08	06.01		
Figure 3*A*		VEH	7	49.77	05.45		*t*_(12)_ = 0.56, *p* = 0.57
		1A-116	7	45.14	06.05		
Figure 3*B*		VEH	7	74.94	06.24		*t*_(7.91)_ = 0.58, *p* = 0.57
		1A-116	8	71.02	02.50		
Figure 3*C*		VEH	8	88.68	03.92		*t*_(13)_ = 0.78, *p* = 0.44
		1A-116	7	82.30	07.49		
Figure 3*D*		VEH	7	36.87	02.95		*t*_(12)_ = 0.60, *p* = 0.55
		1A-116	7	33.09	05.45		
Figure 4*B*		VEH	7	61.39	06.27		*t*_(12)_ = 0.86, *p* = 0.40
		1A-116	7	54.90	04.11		
Figure 4*C*		VEH	7	40.30	05.09		*t*_(12)_ = 1.06, *p* = 0.30
		1A-116	7	50.09	07.68		
Figure 5	VEH	VEH	7	64.46	06.77	ns	*F*_(1,26)_ = 0.14, *p* = 0.70 for interaction *F*_(1,26)_ = 3.82, *p* = 0.06 for intra-CA1 treatment *F*_(1,26)_ = 2.02, *p* = 0.16 for intraperitoneal treatment
		ANI	7	54.48	03.77		
	1A-116	VEH	8	57.85	08.28	ns	
		ANI	8	43.01	05.09		

Total exploration time (s) during test sessions, expressed as mean and SEM. ns, not significant in Sidak’s multiple-comparisons test after two-way ANOVA.

## Results

**Figure 1.**
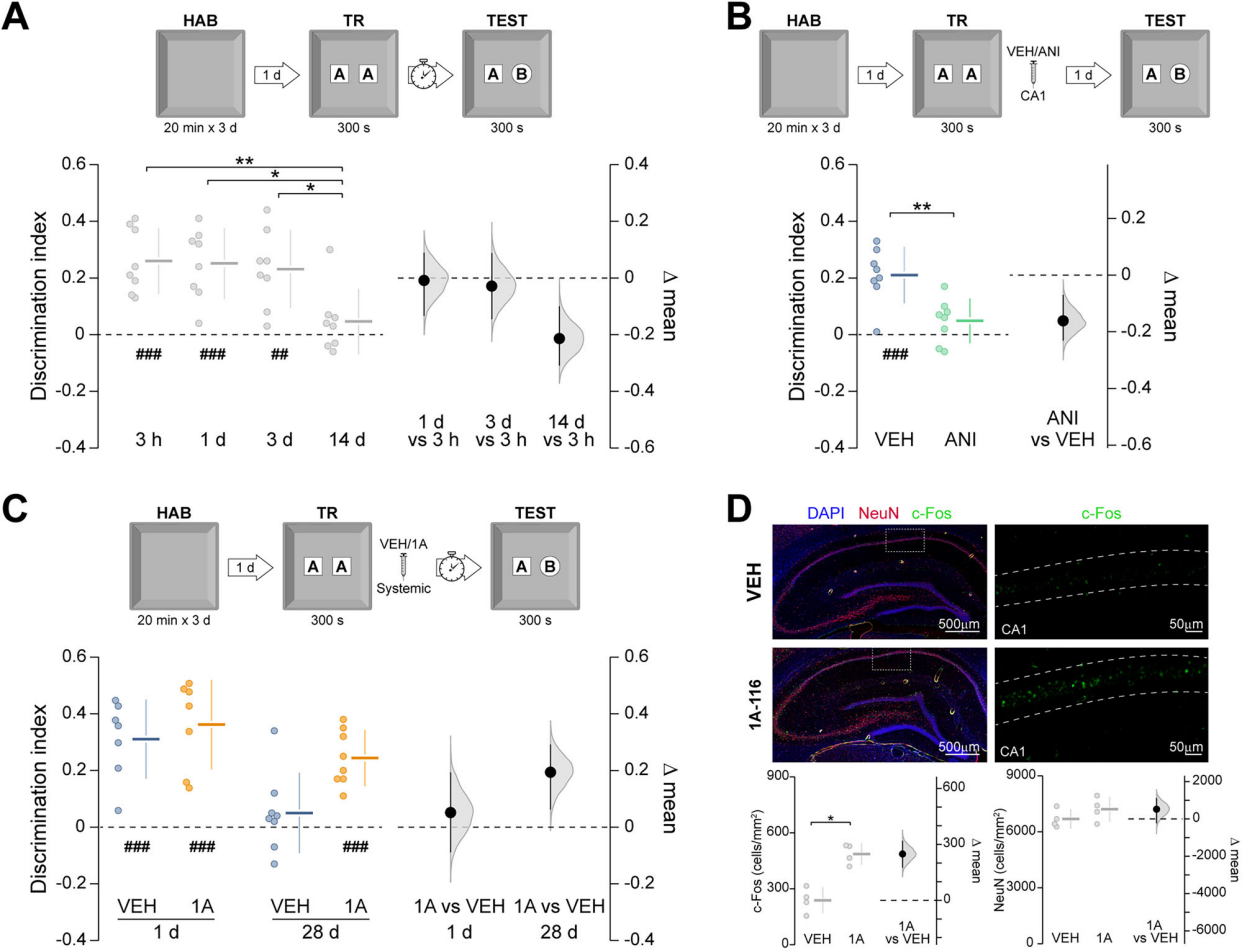
Rac1 inhibition after training enhances ORM retention. ***A***, Young adult male rats underwent daily 20 min habituation sessions (HAB) in the training arena for 3 consecutive days. One day after the final habituation session, the animals were placed in the arena containing two identical novel objects (***A*** and ***A’***) and allowed to explore them freely for 300 s (training session; TR). ORM retention was evaluated 3 h and 1, 3, or 14 d after training by returning the animals to the arena for 300 s in the presence of the familiar object A and a novel object B (TEST). ***B***, Animals were trained as in ***A*** and, 5 min later, received intra-CA1 injections of vehicle (VEH) or anisomycin (ANI; 100 µg/µl; 1 µl/side). ORM retention was evaluated 1 d after training. ***C***, Animals were trained as in ***A*** and, 5 min later, received systemic (i.p.) administration of VEH or 1A-116 (1A; 20 mg/kg). ORM retention was assessed 1 or 28 d after training. ***D***, Naive rats received VEH or 1A-116 and were transcardially perfused with 4% paraformaldehyde (PFA) 90 min later. c-Fos and NeuN expression levels were quantified in CA1 by immunofluorescence. Data are presented as individual data points with mean ± SD (left axes). Estimation plots show the mean difference between groups (black dots), the bootstrapped sampling distribution (gray shading), and the 95% confidence intervals (black error bars). ^##^*p* < 0.01, ^###^*p* < 0.001 in one-sample *t* test (theoretical mean = 0). *p < 0.05, **p < 0.01 in unpaired *t* test, Mann–Whitney test, Tukey’s multiple-comparisons test after one-way ANOVA, or Sidak’s multiple-comparisons test after two-way ANOVA.

Following training in the NOR task, newly formed ORMs exist initially in a transient, hippocampus-independent form, and only those that undergo hippocampus-dependent consolidation persist as long-term representations (Rossato et al., 2007, 2025; Cohen and Stackman, 2015). To confirm this assertion, young adult Wistar rats were habituated to the training arena for 20 min per day over 3 consecutive days. On the fourth day, the animals were exposed to two identical, neutral novel objects for 300 s, producing an ORM that decays within a few days [Fig. 1*A*; *F*_(3,28)_ = 5.51, *p* = 0.004; *p* = 0.008 for 3 h vs 14 d, *p* = 0.011 for 1 d vs 14 d, *p* = 0.025 for 3 d vs 14 d in Tukey's multiple-comparisons test after one-way ANOVA. Mean differences: 1 d vs 3 h = −0.008 (95% CI −0.134, 0.088); 3 d vs 3 h = −0.028 (95% CI −0.145, 0.087); 14 d vs 3 h = −0.214 (95% CI −0.309, −0.1)] and depends on de novo protein synthesis in the dorsal hippocampus [Fig. 1*B*; *t*_(14)_ = 3.67, *p* = 0.002 in unpaired *t* test, ANI vs VEH mean difference = −0.161 (95% CI −0.231, −0.07)].

We then assessed the role of Rac1 in long-term ORM retention. Five minutes after NOR training, rats received intraperitoneal injections of either vehicle or 1A-116 (20 mg/kg). This compound is a selective Rac1 inhibitor that binds to its Trp56 residue and disrupts its interaction with guanine nucleotide exchange factors, including P-Rex1, Vav2, Vav3, and Tiam1, without affecting related GTPases such as Cdc42 (Cardama et al., 2014; González et al., 2020). ORM retention was evaluated 1 or 28 d later by re-exposing the animals to the training arena containing one familiar object and one novel object. Vehicle-treated animals displayed a preference for the novel object 1 d, but not 28 d, after training, whereas 1A-116-treated rats showed preference for the novel object at both timepoints (Fig. 1*C*; *F*_(1,26)_ = 2.11, *p* = 0.157 for interaction; *F*_(1,26)_ = 6.27, *p* = 0.018 for treatment; *F*_(1,26)_ = 15.35, *p* = 0.0006 for test session in two-way ANOVA. Mean differences: 1A-1d vs VEH-1d = 0.051 (95% CI −0.088, 0.193); 1A-28d vs VEH-28d = 0.194 (95% CI 0.062, 0.291)]. In naive rats, systemic 1A-116 increased c-Fos expression in CA1 90 min postinjection [Fig. 1*D*; *U* = 0, *p* = 0.0286 in Mann–Whitney test, 1A vs VEH mean difference = 2.48e + 02 (95% CI 1.76e + 02, 3.18e + 02)], confirming central activity of the compound. These results replicate and extend prior evidence showing that Rac1 inhibition around training enhances long-term ORM retention (Liu et al., 2016; O’Leary et al., 2024).

**Figure 2.**
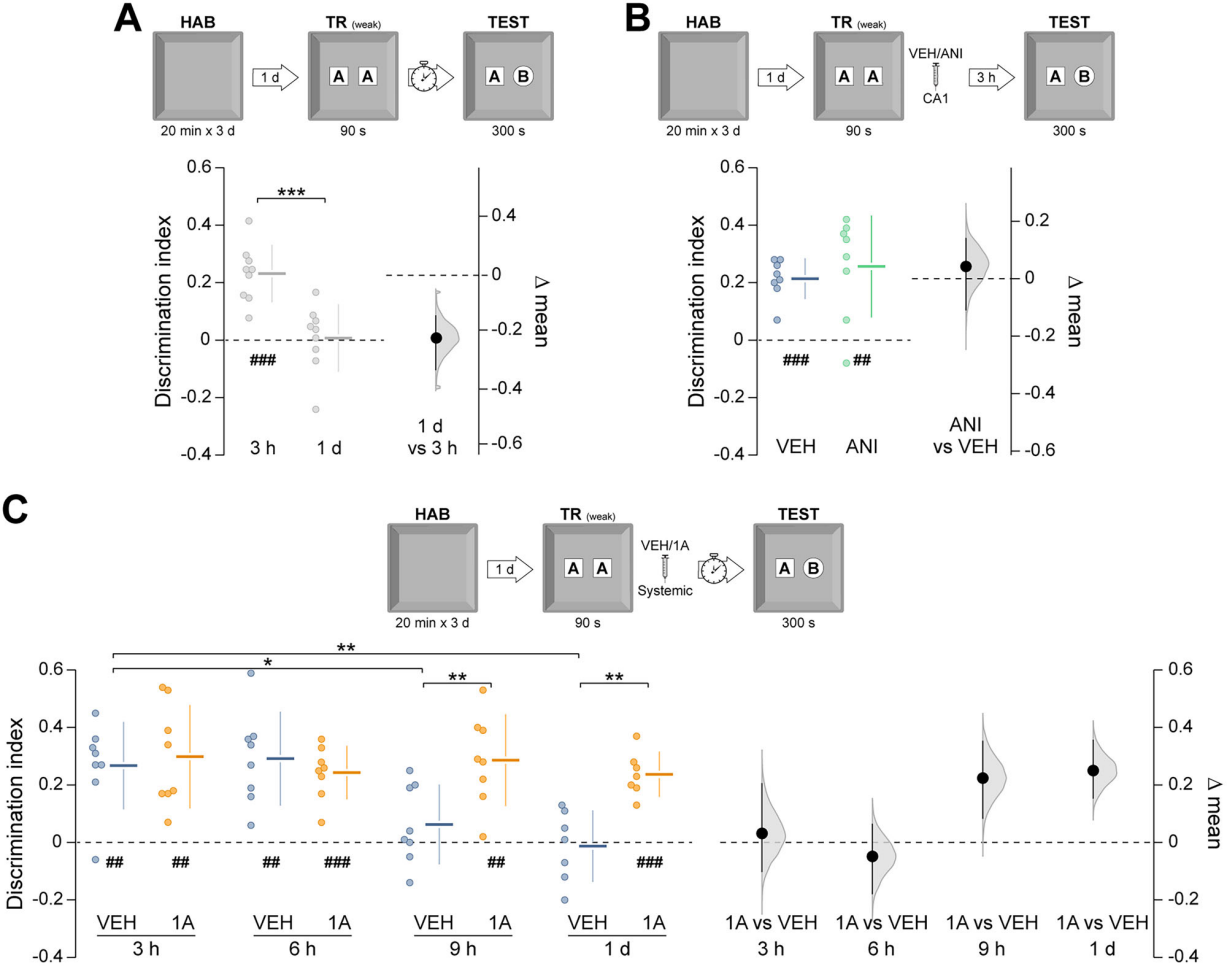
Rac1 inhibition after weak training promotes long-term ORM formation. ***A***, Young adult male rats underwent daily 20 min habituation sessions (HAB) in the training arena for 3 consecutive days. One day after the final habituation session, the animals were placed in the arena containing two identical novel objects (***A*** and ***A’***) and allowed to explore them freely for 90 s (weak training session; TR weak). ORM retention was evaluated 3 h or 1 d after training by returning the animals to the arena for 300 s in the presence of the familiar object A and a novel object B (TEST). ***B***, Animals were trained as in ***A*** and, 5 min later, received intra-CA1 injections of vehicle (VEH) or anisomycin (ANI; 100 µg/µl; 1 µl/side). ORM retention was evaluated 3 h after training. ***C***, Animals were trained as in A and, 5 min later, received systemic (i.p.) administration of VEH or 1A-116 (1A; 20 mg/kg). ORM retention was assessed 3, 6, and 9 h or 1 d after training. Data are presented as individual data points with mean ± SD (left axes). Estimation plots show the mean difference between groups (black dots), the bootstrapped sampling distribution (gray shading), and the 95% confidence intervals (black error bars). ^##^*p* < 0.01, ^###^*p* < 0.001 in one-sample *t* test (theoretical mean = 0). *p < 0.05, **p < 0.01, ***p < 0.001 in unpaired *t* test or Sidak’s multiple-comparisons test after two-way ANOVA.

**Figure 3.**
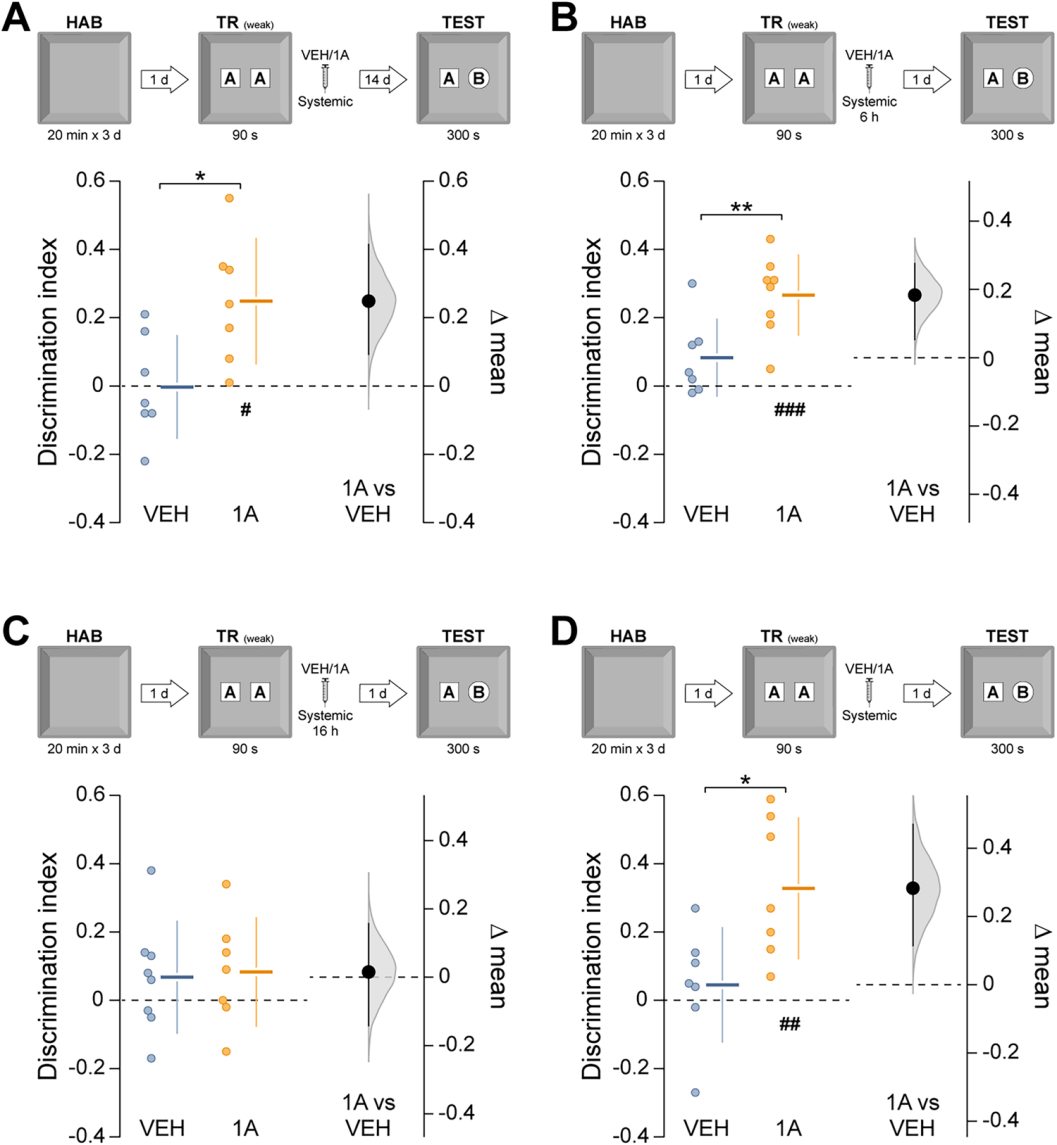
The promnesic effect of Rac 1 inhibition is long-lasting, time-dependent, and age-independent. ***A***, Young adult male rats underwent daily 20 min habituation sessions (HAB) in the training arena for 3 consecutive days. One day after the final habituation session, the animals were placed in the arena containing two identical novel objects (***A*** and ***A’***) and allowed to explore them freely for 90 s (weak training session; TR weak). Five min after training, they received systemic (i.p.) administration of VEH or 1A-116 (1A; 20 mg/kg). ORM retention was evaluated 14 d after training by returning the animals to the arena for 300 s in the presence of the familiar object A and a novel object B (TEST). ***B***, Animals were trained as in ***A*** and received VEH or 1A-116 6 h later. ORM retention was evaluated 1 d after training. ***C***, Animals were trained as in ***A*** and received VEH or 1A-116 16 h later. ORM retention was evaluated 1 d after training. ***D***, Middle-aged rats (14–16 months) were trained as in ***A*** and, 5 min later, received VEH or 1A-116. ORM retention was evaluated 1 d after training. Data are presented as individual data points with mean ± SD (left axes). Estimation plots show the mean difference between groups (black dots), the bootstrapped sampling distribution (gray shading), and the 95% confidence intervals (black error bars).^#^*p* < 0.05, ^##^*p* < 0.01, ^###^*p* < 0.001 in one-sample *t* test (theoretical mean = 0). *p < 0.05, **p < 0.01 in unpaired *t* test.

**Figure 4.**
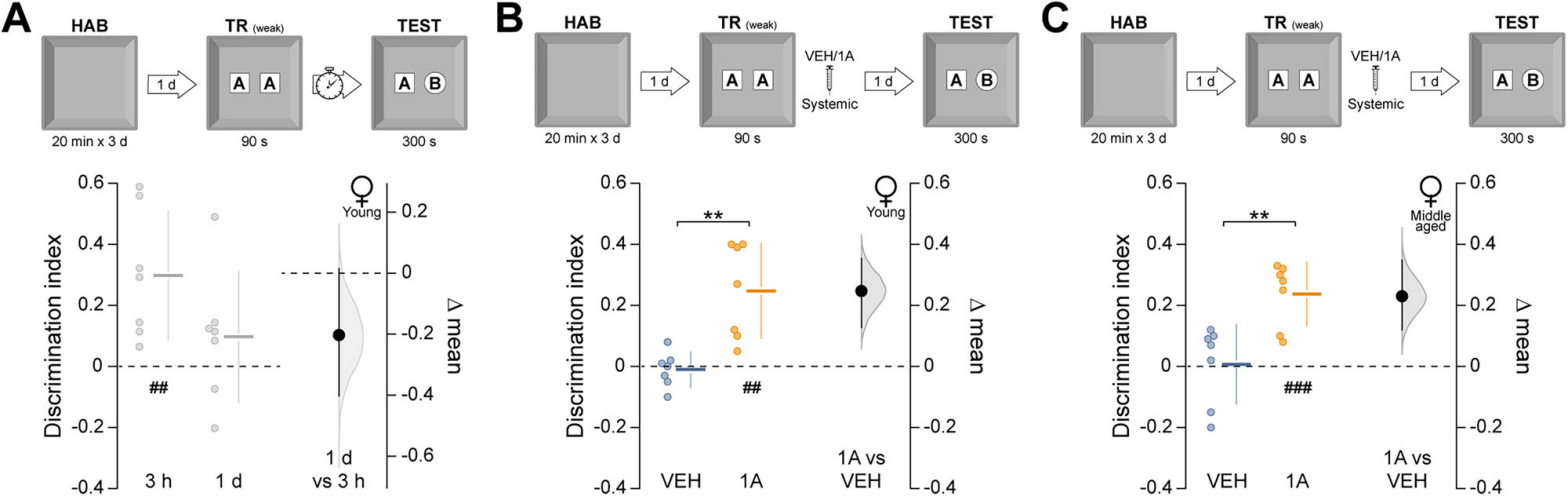
Rac1 inhibition promotes long-term ORM formation in young and middle-aged female rats. ***A***, Young adult female rats underwent daily 20 min habituation sessions (HAB) in the training arena for 3 consecutive days. One day after the final habituation session, the animals were placed in the arena containing two identical novel objects (***A*** and ***A’***) and allowed to explore them freely for 90 s (weak training session; TR weak). ORM retention was evaluated 3 h or 1 d after training by returning the animals to the arena for 300 s in the presence of the familiar object A and a novel object B (TEST). ***B***, Young adult female rats were trained as in ***A*** and, 5 min later, received systemic (i.p.) administration of vehicle (VEH) or 1A-116 (1A; 20 mg/kg). ORM retention was assessed 1 d after training. ***C***, Middle-aged female rats were trained as in A and, 5 min later, received VEH or 1A-116. ORM retention was assessed 1d after training. Data are presented as individual data points with mean ± SD (left axes). Estimation plots show the mean difference between groups (black dots), the bootstrapped sampling distribution (gray shading), and the 95% confidence intervals (black error bars). ^##^*p* < 0.01, ^###^*p* < 0.001 in one-sample *t* test (theoretical mean = 0). **p < 0.01 in unpaired *t* test.

We next asked whether Rac1 inhibition facilitates the formation of long-term ORM after weak learning conditions. Rats underwent a brief 90 s NOR training session, which normally produces a hippocampus-independent short-term ORM that decays within hours [Gonzalez et al., 2019; Fig. 2*A*; *t*_(16)_ = 4.43, *p* = 0.0004 in unpaired *t* test, test-1 d vs test-3 h mean difference = −0.226 (95% CI −0.331, −0.141); Fig. 2*B*]. Five minutes after training, animals received vehicle or 1A-116 and were tested at multiple intervals. Novel object preference declined within 9 h in vehicle-treated animals but persisted in those treated with 1A-116 [Fig. 2*C*; *F*_(3,54)_ = 4.20, *p* = 0.009 for interaction; *F*_(1,54)_ = 10.31, *p* = 0.002 for treatment; *F*_(3,54)_ = 3.74, *p* = 0.016 for test session; *p* = 0.028 for VEH-3 h vs VEH-9 h and *p* = 0.006 for VEH-3 h vs VEH-1d; *p* = 0.009 for VEH-9 h vs 1A-9 h and *p* = 0.005 for VEH-1d vs 1A-1d in Sidak's multiple-comparisons test after two-way ANOVA. Mean differences: 1A-3 h vs VEH-3 h = 0.031 (95% CI −0.102, 0.206); 1A-6 h vs VEH-6 h = −0.048 (95% CI −0.18, 0.065); 1A-9 h vs VEH-9 h = 0.224 (95% CI 0.082, 0.354); 1A-1d vs VEH-1d = 0.25 (95% CI 0.153, 0.357)]. The promnesic effect persisted for at least 14 d [Fig. 3*A*; *t*_(12)_ = 2.81, *p* = 0.015 in unpaired *t* test, VEH vs 1A mean difference = 0.251 (95% CI 0.094, 0.419)], was evident when 1A-116 was given within 6 h [Fig. 3*B*; *t*_(13)_ = 3.08, *p* = 0.008 in unpaired *t* test, VEH vs 1A mean difference = 0.183 (95% CI 0.051, 0.278)] but not when delayed to 16 h post-training (Fig. 3*C*), and was also observed in middle-aged animals [Fig. 3*D*; *t*_(12)_ = 2.82, *p* = 0.015 in unpaired *t* test, VEH vs 1A mean difference = 0.283 (95% CI 0.113, 0.471)]. This effect was also consistent in both young and middle-aged female rats [Fig. 4*B*; *t*_(7.6)_ = 4.11, *p* = 0.003 in unpaired *t* test with Welch's correction, VEH vs 1A mean difference = 0.257 (95% CI 0.136, 0.366); Fig 4*C*; *t*_(12)_ = 3.67, *p* = 0.003 in unpaired *t* test, VEH vs 1A mean difference = 0.23 (95% CI 0.119, 0.35)]. Total exploration times were unaltered by treatment (Table 1). 1A-116 did not affect anxiety-like behavior in the elevated plus maze (Table 2).

**Table 2.** 1A-116 does not alter behavior in the elevated plus maze

		*n*	Mean	SEM	Statistical analysis
Entries open arms (#)	VEH	5	4.2	01.28	*t*_(8)_ = 0.26, *p* = 0.79
	1A-116	5	4.6	0.81	
Time open arms (s)	VEH	5	49.8	22.44	*t*_(8)_ = 0.13, *p* = 0.89
	1A-116	5	46.2	13.64	
Distance open arms (m)	VEH	5	1.68	0.85	*t*_(8)_ = 0.34, *p* = 0.73
	1A-116	5	1.36	0.34	
Total distance (m)	VEH	5	20.88	0.59	*t*_(8)_ = 2.07, *p* = 0.07
	1A-116	5	19.34	0.44	

Animals received systemic (i.p.) administration of vehicle (VEH) or 1A-116 (20 mg/kg), and 1 d later they freely explored the maze for 10 min.

**Figure 5.**
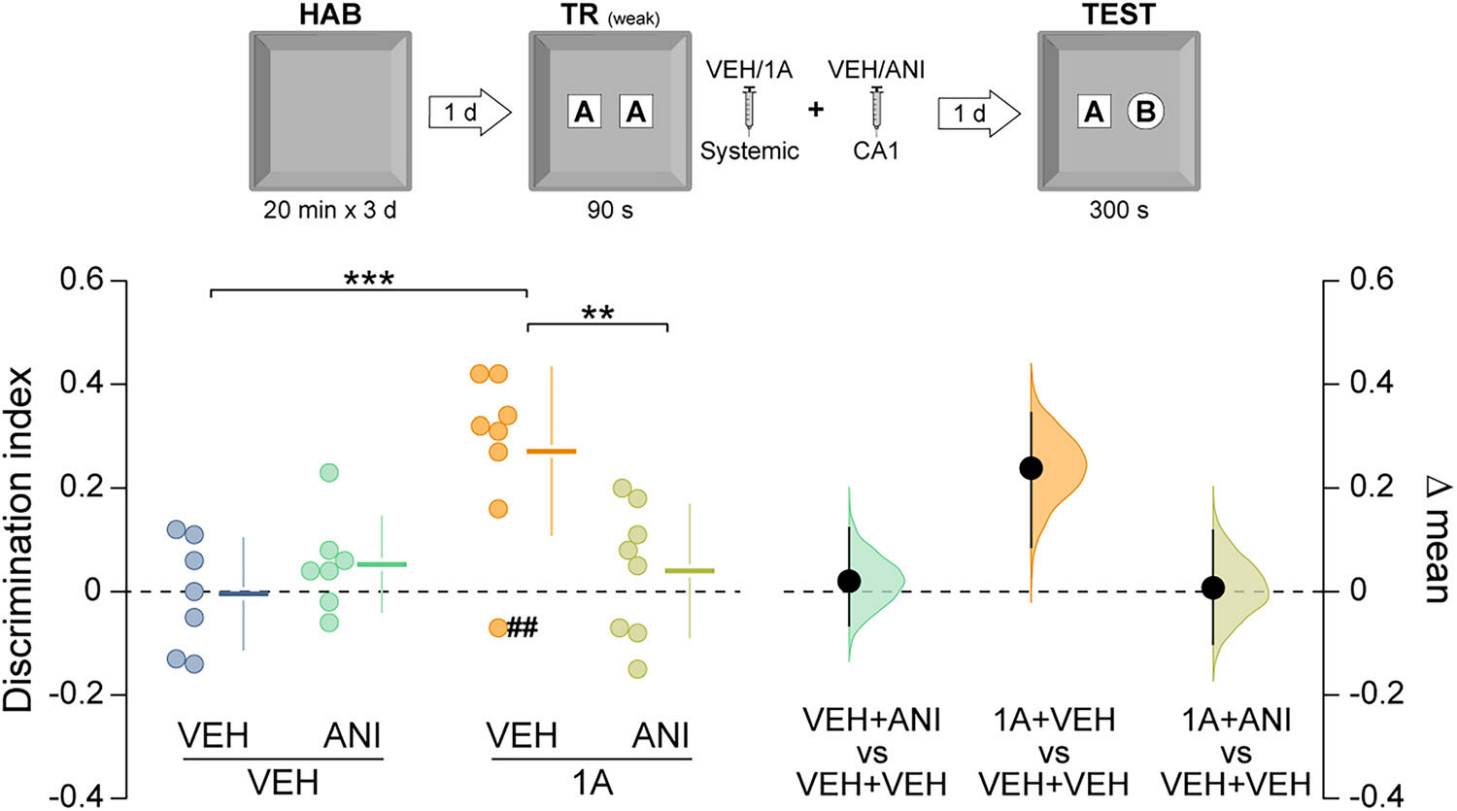
The promnesic effect of Rac 1 inhibition requires hippocampal protein synthesis. Young adult male rats underwent daily 20 min habituation sessions (HAB) in the training arena for 3 consecutive days. One day after the last habituation session, the animals were placed in the arena containing two identical novel objects (***A*** and ***A’***) and allowed to explore them freely for 90 s (weak training session; TR weak). Five minutes after training, the animals received intra-CA1 injections of vehicle (VEH) or anisomycin (ANI; 100 µg/µl; 1 µl/side). Immediately afterward, they received systemic (i.p.) administration of VEH or 1A-116. ORM retention was evaluated 1 d after training by returning the animals to the arena for 300 s in the presence of the familiar object A and a novel object B (TEST). Data are presented as individual data points with mean ± SD (left axes). Estimation plots show the mean difference between groups (black dots), the bootstrapped sampling distribution (gray shading), and the 95% confidence intervals (black error bars). ^##^*p* < 0.01 in one-sample *t* test (theoretical mean = 0). **p < 0.01, ***p < 0.001 in Sidak’s multiple-comparisons test after two-way ANOVA.

To determine whether the long-term ORM induced by 1A-116 requires hippocampal protein synthesis, as in canonical consolidation, rats trained for 90 s in the NOR task received intra-CA1 infusions of the protein synthesis inhibitor anisomycin (100 µg/side) or vehicle 5 min after training. Anisomycin completely blocked the prolonged retention produced by 1A-116 [Fig. 5; *F*_(1,26)_ = 9.71, *p* = 0.004 for interaction; *F*_(1,26)_ = 3.54, *p* = 0.071 for treatment; *F*_(1,26)_ = 8.05, *p* = 0.008 for test session; *p* = 0.0005 for VEH + VEH vs 1A + VEH; *p* = 0.002 for 1A + VEH vs 1A + ANI in Sidak's multiple-comparisons test after two-way ANOVA. Mean differences; VEH + ANI vs VEH + VEH = 0.057 (95% CI −0.03, 0.161); 1A + VEH vs VEH + VEH = 0.276 (95% CI 0.121, 0.383); 1A + ANI vs VEH + VEH = 0.044 (95% CI −0.0659, 0.157)], indicating that Rac1 inhibition promotes hippocampus-dependent memory consolidation.

## Discussion

Systemic post-training inhibition of Rac1 with the selective antagonist 1A-116 significantly enhanced ORM persistence, promoting novelty preference for nearly 1 month without affecting locomotor, exploratory, or anxiety-related behaviors. When combined with a brief, subthreshold training session, Rac1 inhibition converted a transient ORM into a long-lasting, hippocampal protein synthesis-dependent memory. This effect was temporally restricted to the consolidation window, observed across sexes and ages, and abolished by intra-CA1 anisomycin. Taken together, these results suggest that Rac1 activity after learning functions as a molecular constraint on consolidation, rather than driving the active forgetting of ORMs.

Two main findings support this interpretation. First, Rac1 inhibition converted a weak, hippocampus-independent short-term memory into a long-lasting trace that, like canonical long-term ORM formation, required post-training de novo protein synthesis in dorsal CA1 (Rossato et al., 2007; Myskiw et al., 2008). The suppression of the 1A-116 effect by anisomycin suggests that Rac1 inhibition allows the engagement of hippocampal consolidation mechanisms that would otherwise not be recruited by weak training. Thus, Rac1 appears to regulate whether newly encoded information remains transient or progresses toward long-term stabilization. Second, the promnesic effect of 1A-116 emerged several hours after weak training and persisted for at least 14 d. This temporal profile is inconsistent with a forgetting-attenuation account, which would be expected to slow down the decay of the original memory trace while preserving its independence from consolidation blockers (Shuai et al., 2010).

Our work expands the current understanding of Rac1's contribution to memory processes. Earlier work in *Drosophila* linked Rac1 primarily to active forgetting, where its activation promotes memory decay and its suppression prolongs short-term traces (Shuai et al., 2010; Zhang et al., 2018). In contrast, we found that post-training Rac1 inhibition in rats enables the emergence of a protein synthesis-dependent long-term memory, suggesting a fundamental difference between prolonging a labile trace and enabling full consolidation. At least for ORM in rats, Rac1 thus appears to regulate the consolidation threshold, influencing whether newly encoded information is stabilized as a durable engram.

Mechanistically, Rac1 acts at the intersection of actin dynamics and translational control (Feuge et al., 2019; Triantopoulou and Vidaki, 2022), modulating actin polymerization through the PAK-LIMK-cofilin cascade and leading to dendritic spine destabilization and reversal of potentiation (Oh et al., 2010; Liu et al., 2016). Rac1 also interacts with the ERK1/2 and mTOR pathways (Durán and Hall, 2012; Bachmann et al., 2013) that regulate translational activation during consolidation (Myskiw et al., 2008; Medina and Viola, 2018). We propose that Rac1 normally constrains protein synthesis required for memory stabilization, thereby setting a consolidation threshold through coordination of cytoskeletal remodeling and translational activity after training. Under weak training conditions, transient Rac1 activation may allow short-term ORM formation while limiting recruitment of translation-dependent processes necessary for long-term stabilization. In this context, inhibiting Rac1 lifts this constraint, enabling protein synthesis for durable storage. In stronger training conditions, activity-dependent regulatory mechanisms, possibly involving molecules such as α2-chimaerin (Jiang et al., 2016; Lv et al., 2019), may naturally downregulate Rac1 signaling to permit consolidation. Consistent with this, Tiam1, an upstream regulator of Rac1, constrains memory storage by limiting NMDAR-mediated hippocampal plasticity, and strong neuronal activity induces degradation of Tiam1, relieving this restriction (Blanco et al., 2024). More broadly, several negative regulators of synaptic plasticity, including suppressor genes, phosphatase activity, and perineuronal nets, serve as regulatory checkpoints during consolidation, determining whether learning-induced facilitation is stabilized and, consequently, how long a memory persists (Abel and Kandel, 1998; Reichelt et al., 2019; Carulli and Verhaagen, 2021; Foley et al., 2021).

Our results are also compatible with the synaptic tagging and protein capture hypothesis (Frey and Morris, 1997), which explains the transition of short-term to long-term memories (Moncada and Viola, 2007). In this view, a weak training induces transient synaptic tags but fails to produce or recruit sufficient plasticity-related proteins for long-term stabilization. Rac1 could function as a regulator of the tagging–capture balance, where its activation limits the persistence of synaptic tags or restricts protein availability by limiting actin stabilization and translational signaling. Inhibiting Rac1 may therefore extend the tagging window or enhance protein capture, allowing weakly activated synapses to stabilize and support long-term memory storage.

However, it must be borne in mind that although 1A-116 administration was effective in promoting long-term ORM, it likely influenced multiple brain regions beyond the hippocampus. Future studies should determine how Rac1 inhibition affects specific circuits or cell types using localized infusions or genetic approaches. Identifying the intermediate signaling molecules that connect Rac1 to protein synthesis, including PAK, LIMK, and mTOR, will be important for establishing a detailed causal chain. Testing Rac1 modulation in other forms of memory such as spatial, fear, or extinction paradigms would also help determine whether its role as a consolidation brake generalizes across mnemonic systems. Furthermore, consolidation and forgetting are not mutually exclusive processes and can operate in parallel after learning. While de novo protein synthesis in different brain regions is required for ORMs consolidation (Lima et al., 2009; Cohen and Stackman, 2015; Rossato et al., 2019), accumulating evidence indicates that active forgetting can also engage protein synthesis-dependent mechanisms. Thus, although our findings strongly support a role for Rac1 in regulating the consolidation threshold of ORMs, they do not exclude the possibility that Rac1 also contributes to active forgetting under different temporal windows or training conditions. It is also possible that Rac1 exerts region-specific and partially overlapping effects, constraining consolidation in some brain areas while facilitating forgetting in others, and that the methodological tools we employed lack the resolution necessary to reveal these distinct contributions.

From a translational perspective, Rac1 overactivation has been associated with cognitive decline and memory deficits during aging, as well as with neurodegenerative disorders such as Alzheimer's disease, in which recognition memory is particularly affected (Wu et al., 2019; Kaushik et al., 2022). Because 1A-116 is a brain-permeable, selective Rac1 inhibitor with a favorable safety profile (Cardama et al., 2022), our findings suggest that targeting Rac1 may help mitigate declarative memory impairment in dementia and related disorders.

In summary, Rac1 activity after learning functions as a molecular constraint that determines whether newly acquired episodic-like information is transient or stabilized into long-term memory, helping prevent the over-stabilization of weak or irrelevant experiences and supporting memory flexibility. This work reframes Rac1 as a key regulator of the consolidation threshold, providing a mechanistic explanation for how cytoskeletal signaling influences the persistence of memory traces and identifying Rac1 as a promising target for cognitive enhancement.

## Synthesis

Reviewing Editor: Timothy Jarome, Virginia Polytechnic Institute and State University Decisions are customarily a result of the Reviewing Editor and the peer reviewers coming together and discussing their recommendations until a consensus is reached. When revisions are invited, a fact-based synthesis statement explaining their decision and outlining what is needed to prepare a revision will be listed below. The following reviewer(s) agreed to reveal their identity: Ryan Parsons, Grace Schenatto Pereira Moraes.

This study is novel and relevant to the field, with results clearly presented. However, several concerns were raised that need addressed

1. The manuscript relies primarily on one-sample t-tests of discrimination index and exploration time data to determine if each group was different than zero, rather than using group comparisons. Without direct group comparisons, the effectiveness of any experimental manipulation cannot be determined. It is not appropriate to conclude that a manipulation is effective by only testing if one group is different than zero without comparing them directly.

2. The authors provide tests which help distinguish an effect of Rac1 inhibition on memory consolidation vs preventing forgetting. These are reasonable tests on the surface, but the results are somewhat equivocal given that there is evidence that neural mechanisms of forgetting are engaged upon initial learning on a similar time course to those thought to be involved in consolidation of memory.

3. Figure 2 shows that a weak memory lasted up to 6 h and was independent of hippocampal protein synthesis. In that case, this memory is irrelevant and forgetting processes are high. Inhibiting Rac1 prolongs memory (9h, 1d). This demonstrates a clear impact on preventing forgetting, although the authors mainly discuss consolidation. On the other hand, if Rac1 activity is restricted to forgetting processes, it would be expected to be high in the 90s protocol and for the effect of 1A to be present; otherwise, the protocol's relevance was not properly controlled. However, 1A has no effect on ORM tested 3h and 6h after training. Does Rac1 not act on ORM forgetting? It would be useful for the authors to discuss these results in more detail. In fact, the authors tangentially argued that short- and long-term memories are distinct; however, it would be highly informative for readers to elaborate on these concepts.

4. Figure 1 confirmed that 300s training produces a memory lasting 3 days but not 14 days, and that this memory depends on hippocampal protein synthesis to persist for 1 day. Why did the authors choose to test the effect of 1A on 28-day memory? Could it be that inhibiting Rac-1 does not lead to forgetting a 1-day memory because, as a strong memory, it requires more molecular processes or rounds for forgetting to occur?

5. The intriguing results show that 1A, administered immediately after 90s training, promotes ORM tested at 9h, 1 day (figure 2), and 14 days (figure 3). The effect of 1A was maintained when injected 6h after training but not 16h. Therefore, it seems this drug acts within a 6h time window, but its effects are observable only when memory is tested late. Is this a demonstration of STM and LTM distinction rather than a distinct effect of Rac1 in consolidation and active forgetting? Does the fact that a 6h injection still prevents forgetting of a weak memory reinforce the idea of Rac1's role in forgetting? The lack of effect of 1A 16h after training may mean that, at this time point, the processes of forgetting have already occurred?

6. In Figure 5, the authors showed that after 90s of training, a 1-day memory is forgotten. Inhibiting Rac1 prevented this from happening. This process depends on protein synthesis. An alternative discussion should consider whether protein synthesis is required for active forgetting.

7. Based on the results, the following statements should be better explained and backed by more references to support the authors' conclusions:

a. "In contrast, Rac1 inhibition in rats enables the emergence of a protein synthesis dependent long-term memory, suggesting a fundamental difference between prolonging a labile trace and triggering full consolidation. This evidence supports the idea that Rac1 regulates the fate of newly encoded information by determining whether it remains transient or becomes stabilized as a durable engram."

b. "This framework positions Rac1 as a molecular brain on consolidation, similar to other systems that limit synaptic plasticity, such as phosphatase activity or perineuronal nets (Reichelt et al., 2019; Foley et al., 2021; Carulli and Verhaagen, 2021)."

c. "Our findings are also consistent with the view that short- and long-term memories represent distinct, yet coexisting processes triggered by a single learning event (Izquierdo et al., 1998). Rac1 may operate as a molecular switch that adjusts the balance between these processes. When Rac1 activity is high, memories remain transient. When Rac1 is inhibited, consolidation mechanisms are engaged, and stable, protein synthesis-dependent memories emerge."

8. Please answer these methodological questions

1. Were animals tested only once?

2. Why use ketamine combined with isoflurane? This combination is unusual.

3. Please provide more details on preparing anisomycin.

4. Please explain what you mean by "low light." It would be very useful for reproducibility to report the measurement in lux. For the same reason, please provide more details about the size of the objects and which solution was used to clean the arena and objects.

5. Why did you choose 10s as the cut time for excluding animals? How many animals explore objects for less than 10s?

6. Please provide more details about the mechanisms of action of 1A-116.

## References

[B1] Abel T, Kandel E (1998) Positive and negative regulatory mechanisms that mediate long-term memory storage. Brain Res Brain Res Rev 26:360–378. 10.1016/S0165-0173(97)00050-7 9651552

[B2] Aggleton JP, Nelson AJD (2020) Distributed interactive brain circuits for object-in-place memory: a place for time? Brain Neurosci Adv 4:2398212820933471. 10.1177/2398212820933471 32954003 PMC7479857

[B3] Bachmann VA, Riml A, Huber RG, Baillie GS, Liedl KR, Valovka T, Stefan E (2013) Reciprocal regulation of PKA and Rac signaling. Proc Natl Acad Sci U S A 110:8531–8536. 10.1073/pnas.1215902110 23657011 PMC3666698

[B4] Berg S, et al. (2019) Ilastik: interactive machine learning for (bio)image analysis. Nat Methods 16:1226–1232. 10.1038/s41592-019-0582-9 31570887

[B5] Blanco FA, Saifullah MAB, Cheng JX, Abella C, Scala F, Firozi K, Niu S, Park J, Chin J, Tolias KF (2024) Targeting Tiam1 enhances hippocampal-dependent learning and memory in the adult brain and promotes NMDA receptor-mediated synaptic plasticity and function. J Neurosci 45:e0298242024. 10.1523/JNEUROSCI.0298-24.2024 PMC11800756 39725519

[B6] Cardama GA, Comin MJ, Hornos L, Gonzalez N, Defelipe L, Turjanski AG, Alonso DF, Gomez DE, Menna PL (2014) Preclinical development of novel Rac1-GEF signaling inhibitors using a rational design approach in highly aggressive breast cancer cell lines. Anticancer Agents Med Chem 14:840–851. 10.2174/18715206113136660334 24066799 PMC4104455

[B7] Cardama GA, et al. (2022) Preclinical efficacy and toxicology evaluation of RAC1 inhibitor 1A-116 in human glioblastoma models. Cancers (Basel) 14:4810. 10.3390/cancers14194810 36230732 PMC9562863

[B8] Carulli D, Verhaagen J (2021) An extracellular perspective on CNS maturation: perineuronal nets and the control of plasticity. Int J Mol Sci 22:2434. 10.3390/ijms22052434 33670945 PMC7957817

[B9] Cohen SJ, Stackman RW Jr (2015) Assessing rodent hippocampal involvement in the novel object recognition task: a review. Behav Brain Res 285:105–117. 10.1016/j.bbr.2014.08.002 25169255 PMC7008635

[B10] Cui D, Jiang X, Chen M, Sheng H, Shao D, Yang L, Guo X, Wang Y, Lai B, Zheng P (2021) Activation of Rac1 has an opposing effect on induction and maintenance of long-term potentiation in hippocampus by acting on different kinases. Front Mol Neurosci 14:720371. 10.3389/fnmol.2021.720371 34531724 PMC8438208

[B11] Dalto JF, Medina JH, Pastor V (2025) Molecular underpinnings of memory persistence and forgetting. J Neurochem 169:e70089. 10.1111/jnc.70089 40411122

[B12] Davis RL, Zhong Y (2017) The biology of forgetting—a perspective. Neuron 95:490–503. 10.1016/j.neuron.2017.05.039 28772119 PMC5657245

[B13] Durán RV, Hall MN (2012) Regulation of TOR by small GTPases. EMBO Rep 13:121–128. 10.1038/embor.2011.257 22240970 PMC3271343

[B14] Ennaceur A, Delacour J (1988) A new one-trial test for neurobiological studies of memory in rats. 1: behavioral data. Behav Brain Res 31:47–59. 10.1016/0166-4328(88)90157-X 3228475

[B15] Feuge J, Scharkowski F, Michaelsen-Preusse K, Korte M (2019) FMRP modulates activity-dependent spine plasticity by binding Cofilin1 mRNA and regulating localization and local translation. Cereb Cortex 29:5204–5216. 10.1093/cercor/bhz059 30953439

[B16] Foley K, McKee C, Nairn AC, Xia H (2021) Regulation of synaptic transmission and plasticity by protein phosphatase 1. J Neurosci 41:3040–3050. 10.1523/JNEUROSCI.2026-20.2021 33827970 PMC8026358

[B17] Frey U, Morris RG (1997) Synaptic tagging and long-term potentiation. Nature 385:533–536. 10.1038/385533a0 9020359

[B18] Gonzalez MC, Rossato JI, Radiske A, Pádua Reis M, Cammarota M (2019) Recognition memory reconsolidation requires hippocampal Zif268. Sci Rep 9:16620. 10.1038/s41598-019-53005-8 31719567 PMC6851087

[B19] Gonzalez MC, Rossato JI, Radiske A, Bevilaqua LRM, Cammarota M (2021) Dopamine controls whether new declarative information updates reactivated memories through reconsolidation. Proc Natl Acad Sci U S A 118:e2025275118. 10.1073/pnas.2025275118 34253612 PMC8307459

[B20] Gonzalez MC, Radiske A, Rossato JI, Conde-Ocazionez S, Bevilaqua LRM, Cammarota M (2022) Optogenetic inactivation of the medial septum impairs long-term object recognition memory formation. Mol Brain 15:50. 10.1186/s13041-022-00938-3 35672792 PMC9172102

[B21] González N, Cardama GA, Chinestrad P, Robles-Valero J, Rodríguez-Fdez S, Lorenzo-Martín LF, Bustelo XR, Lorenzano Menna P, Gomez DE (2020) Computational and in vitro pharmacodynamics characterization of 1A-116 Rac1 inhibitor: relevance of Trp56 in its biological activity. Front Cell Dev Biol 8:240. 10.3389/fcell.2020.00240 32351958 PMC7174510

[B22] Haditsch U, Leone DP, Farinelli M, Chrostek-Grashoff A, Brakebusch C, Mansuy IM, McConnell SK, Palmer TD (2009) A central role for the small GTPase Rac1 in hippocampal plasticity and spatial learning and memory. Mol Cell Neurosci 41:409–419. 10.1016/j.mcn.2009.04.005 19394428 PMC2705331

[B23] Ho J, Tumkaya T, Aryal S, Choi H, Claridge-Chang A (2019) Moving beyond *p* values: data analysis with estimation graphics. Nat Methods 16:565–566. 10.1038/s41592-019-0470-3 31217592

[B24] Jiang L, Mao R, Zhou Q, Yang Y, Cao J, Ding Y, Yang Y, Zhang X, Li L, Xu L (2016) Inhibition of Rac1 activity in the hippocampus impairs the forgetting of contextual fear memory. Mol Neurobiol 53:1247–1253. 10.1007/s12035-015-9093-6 25613020

[B25] Kaushik M, Kaushik P, Parvez S (2022) Memory related molecular signatures: the pivots for memory consolidation and Alzheimer’s related memory decline. Ageing Res Rev 76:101577. 10.1016/j.arr.2022.101577 35104629

[B26] Lima RH, Rossato JI, Furini CR, Bevilaqua LR, Izquierdo I, Cammarota M (2009) Infusion of protein synthesis inhibitors in the entorhinal cortex blocks consolidation but not reconsolidation of object recognition memory. Neurobiol Learn Mem 91:466–472. 10.1016/j.nlm.2008.12.009 19141326

[B27] Liu Y, Du S, Lv L, Lei B, Shi W, Tang Y, Wang L, Zhong Y (2016) Hippocampal activation of Rac1 regulates the forgetting of object recognition memory. Curr Biol 26:2351–2357. 10.1016/j.cub.2016.06.056 27593377

[B28] Lv L, Liu Y, Xie J, Wu Y, Zhao J, Li Q, Zhong Y (2019) Interplay between α2-chimaerin and Rac1 activity determines dynamic maintenance of long-term memory. Nat Commun 10:5313. 10.1038/s41467-019-13236-9 31757963 PMC6876637

[B29] Medina JH (2018) Neural, cellular and molecular mechanisms of active forgetting. Front Syst Neurosci 12:3. 10.3389/fnsys.2018.00003 29467630 PMC5808127

[B30] Medina JH, Viola H (2018) ERK1/2: a key cellular component for the formation, retrieval, reconsolidation and persistence of memory. Front Mol Neurosci 11:361. 10.3389/fnmol.2018.00361 30344477 PMC6182090

[B31] Moncada D, Viola H (2007) Induction of long-term memory by exposure to novelty requires protein synthesis: evidence for a behavioral tagging. J Neurosci 27:7476–7481. 10.1523/JNEUROSCI.1083-07.2007 17626208 PMC6672624

[B32] Myskiw JC, Rossato JI, Bevilaqua LR, Medina JH, Izquierdo I, Cammarota M (2008) On the participation of mTOR in recognition memory. Neurobiol Learn Mem 89:338–351. 10.1016/j.nlm.2007.10.002 18039584

[B33] Oh D, et al. (2010) Regulation of synaptic Rac1 activity, long-term potentiation maintenance, and learning and memory by BCR and ABR Rac GTPase-activating proteins. J Neurosci 30:14134–14144. 10.1523/JNEUROSCI.1711-10.2010 20962234 PMC5076888

[B34] O’Leary JD, Bruckner R, Autore L, Ryan TJ (2024) Natural forgetting reversibly modulates engram expression. eLife 12:RP92860. 10.7554/eLife.92860 39499054 PMC11537488

[B35] Paxinos GW, Watson C (2007) *The rat brain in stereotaxic coordinates*, Ed 6. San Diego: Academic.

[B36] Radiske A, Rossato JI, Gonzalez MC, Köhler CA, Bevilaqua LR, Cammarota M (2017) BDNF controls object recognition memory reconsolidation. Neurobiol Learn Mem 142:79–84. 10.1016/j.nlm.2017.02.018 28274823

[B37] Reichelt AC, Hare DJ, Bussey TJ, Saksida LM (2019) Perineuronal nets: plasticity, protection, and therapeutic potential. Trends Neurosci 42:458–470. 10.1016/j.tins.2019.04.003 31174916

[B38] Rossato JI, Bevilaqua LR, Myskiw JC, Medina JH, Izquierdo I, Cammarota M (2007) On the role of hippocampal protein synthesis in the consolidation and reconsolidation of object recognition memory. Learn Mem 14:36–46. 10.1101/lm.422607 17272651 PMC1838544

[B39] Rossato JI, Gonzalez MC, Radiske A, Apolinário G, Conde-Ocazionez S, Bevilaqua LR, Cammarota M (2019) PKMζ inhibition disrupts reconsolidation and erases object recognition memory. J Neurosci 39:1828–1841. 10.1523/JNEUROSCI.2270-18.2018 30622166 PMC6407297

[B40] Rossato JI, Radiske A, Gonzalez MC, Bevilaqua LRM, Cammarota M (2022) On the effect of hippocampal c-Jun N-terminal kinase inhibition on object recognition memory. Front Behav Neurosci 16:1052124. 10.3389/fnbeh.2022.1052124 36578877 PMC9790984

[B41] Rossato JI, Gonzalez MC, Apolinário G, Radiske A, Brisa E, Carneiro LM, Cammarota M (2025) Hippocampal CaMKII regulates the consolidation of recognition memory. Eur J Neurosci 61:e70049. 10.1111/ejn.70049 40029612

[B42] Rossato JI, Ribeiro L, Lima-Silva T, Araujo R, Orvate R, Azevedo AL, Bevilaqua Cammarota N, Baracho AL, Carneiro L, Cammarota M (2026) Upstream CB1R regulation of β-adrenergic memory consolidation. Hippocampus 36:e70084. 10.1002/hipo.70084 41689307 PMC12905559

[B43] Shuai Y, Lu B, Hu Y, Wang L, Sun K, Zhong Y (2010) Forgetting is regulated through Rac activity in *Drosophila*. Cell 140:579–589. 10.1016/j.cell.2009.12.044 20178749

[B44] Tolias KF, Bikoff JB, Burette A, Paradis S, Harrar D, Tavazoie S, Weinberg RJ, Greenberg ME (2005) The Rac1-GEF Tiam1 couples the NMDA receptor to the activity-dependent development of dendritic arbors and spines. Neuron 45:525–538. 10.1016/j.neuron.2005.01.024 15721239

[B45] Triantopoulou N, Vidaki M (2022) Local mRNA translation and cytoskeletal reorganization: mechanisms that tune neuronal responses. Front Mol Neurosci 15:949096. 10.3389/fnmol.2022.949096 35979146 PMC9376447

[B46] Wiens KM, Lin H, Liao D (2005) Rac1 induces the clustering of AMPA receptors during spinogenesis. J Neurosci 25:10627–10636. 10.1523/JNEUROSCI.1947-05.2005 16291935 PMC6725855

[B47] Wu W, et al. (2019) Inhibition of Rac1-dependent forgetting alleviates memory deficits in animal models of Alzheimer’s disease. Protein Cell 10:745–759. 10.1007/s13238-019-0641-0 31321704 PMC6776562

[B48] Zhang X, Li Q, Wang L, Liu ZJ, Zhong Y (2018) Active protection: learning-activated Raf/MAPK activity protects labile memory from Rac1-independent forgetting. Neuron 98:142–155. 10.1016/j.neuron.2018.02.025 29551489

